# Supplementary material for: A Capillary Electrophoresis Method Based on Molecularly Imprinted Solid-Phase Extraction for Selective and Sensitive Detection of Histamine in Foods
Source: Molecules. 2022 Oct 17;27(20):6987. doi: 10.3390/molecules27206987 (PMC9610981; doi:10.3390/molecules27206987)
Supplement: Supplementary file 1 [file molecules-27-06987-s001.zip › molecules-1956865-supplementary (1)-done.pdf]

molecules

MDPI

A Selective and Sensitive Method for the Detection of Histamine in Foods by Capillary Electrophoresis Coupled with Molecularly Imprinted Solid-Phase Extraction

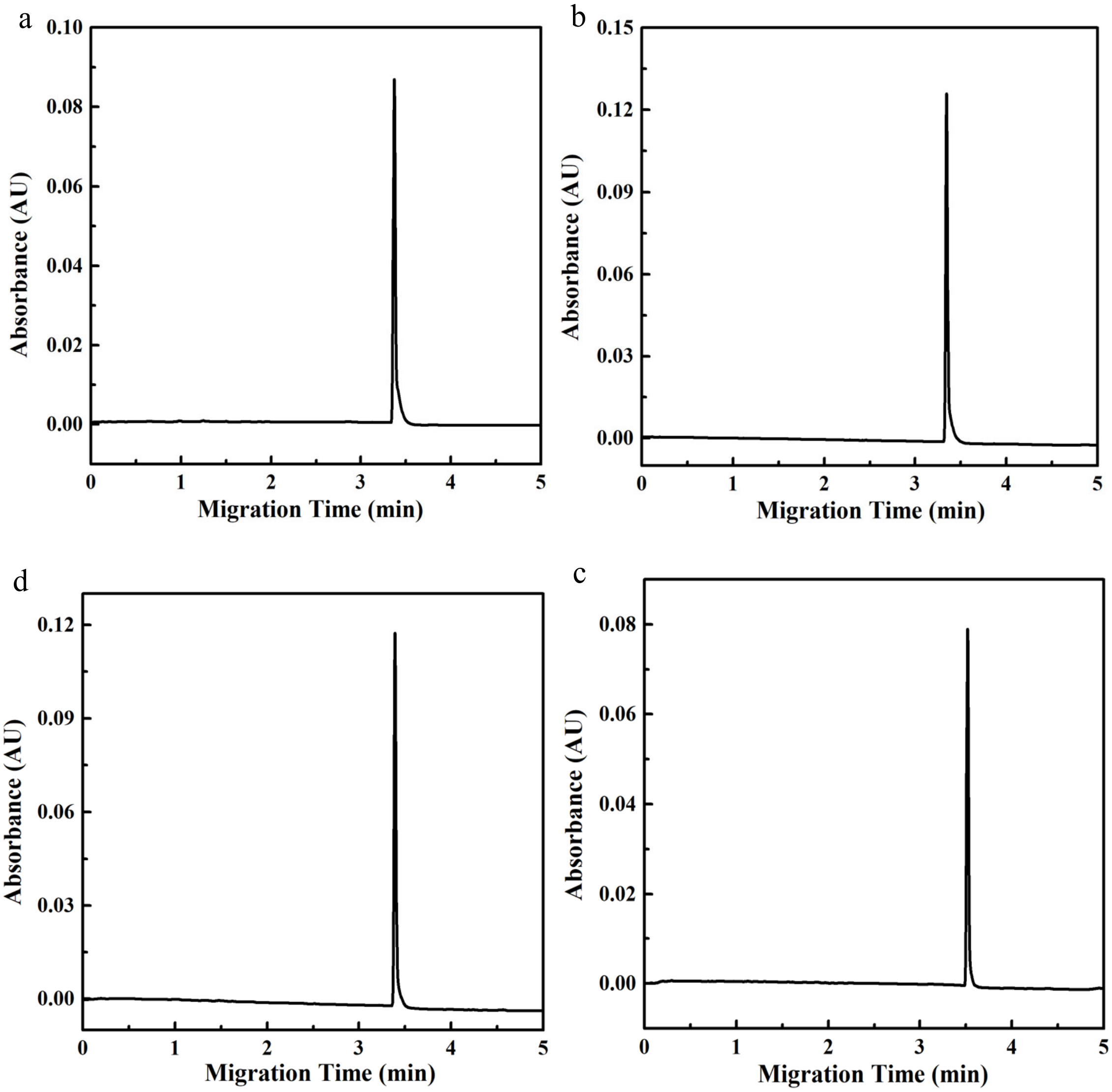

Figure S1. The electropherograms of histamine detection in the (a) soy sauce, (b) fish, (c) pork and (d) prawn samples.

Table S1. The results of CE and HPLC analysis for histamine detection in the samples (n = 3).

Samples	Found Level of MISPE-CE （mg/L ± RSD）	Found Level of HPLC （mg/L ± SD）
Fish	2.51 ± 2.30	2.50 ± 0.16
Prawn	2.22 ± 4.40	2.24 ± 0.01
Pork	0.51 ± 0.19	0.51 ± 0.13
Chicken breast	ND[a]	ND
Soy sauce	1.47 ± 7.63	1.49 ± 0.10

[a] ND: not detected.

Table S2. Comparison of the developed MISPE-CE method with previous reported methods for the determination of histamine.

Detector	Method Used	Linearity (μg/L)	LOD (μg/L)	Ref.
DAD	CE	5.0×10^3-2.0×10^5	2.0×10^3	[35]
UV	cITP-CZE-COND	22-222	4.0	[36]
DAD	CE by field-amplified sample stacking and in-capillary derivatization	5.6×10^4-1.1×10^8	3.7×10^2	[37]
UV	cITP-CZE	2.0×10^2-1.0×10^4	3.5×10^2	[38]
UV	CZE	1.0×10^3-1.0×10^5	4.8×10^2	[39]
DAD	MISPE	0.1-100.0	0.087	This work
